# Aktuelles zur Herz-Nieren-Interaktion: Zusammenfassung wichtiger klinischer Studien aus 2020

**DOI:** 10.1007/s00059-021-05043-0

**Published:** 2021-06-09

**Authors:** Insa E. Emrich, Mert Tokcan, Hussam Al Ghorani, Vedat Schwenger, Felix Mahfoud

**Affiliations:** 1 Klinik für Innere Medizin III, Klinik für Kardiologie, Angiologie und Internistische Intensivmedizin, IMED, Universitätsklinikum des Saarlandes, Homburg, Deutschland; 2 Klinik für Nieren‑, Hochdruck- und Autoimmunerkrankungen, Transplantationszentrum Stuttgart, Klinikum der Landeshauptstadt Stuttgart gKAöR, Stuttgart, Deutschland

**Keywords:** Chronische Nierenerkrankung, Kardiovaskuläre Morbidität, SGLT2-Inhibitoren, Mineralokortikoidrezeptorantagonisten, Nierenersatztherapie, Chronic renal insufficiency, Cardiovascular morbidity, SGLT2 inhibitors, Mineralocorticoid receptor antagonists, Renal replacement therapy

## Abstract

Chronisch nierenkranke Patienten weisen eine erhöhte kardiovaskuläre Morbidität und Sterblichkeit auf. Im letzten Jahr sind einige wichtige Studien zur Herz-Nieren-Interaktion veröffentlicht worden, die im Folgenden zusammengefasst und diskutiert werden. In der DAPA-CKD-Studie sowie in der SCORED-Studie konnten 2 unterschiedliche SGLT2(„sodium-glucose linked transporter 2“)-Inhibitoren (Dapagliflozin und Sotagliflozin) die Prognose von chronisch nierenkranken Patienten mit und ohne Diabetes nachweislich verbessern. Auch die Ergebnisse der randomisierten Studie zum neuen Mineralokortikoidrezeptorantagonisten Finerenon – FIDELIO-DKD – liefern einen vielversprechenden neuen Therapieansatz für Patienten mit diabetischer Nephropathie. Die veröffentlichten Daten der ISCHEMIA-CKD-Studie bei Patienten mit koronarer Herzkrankheit und Untersuchungen zum Einfluss einer TAVI („transcatheter aortic valve implantation“) auf die Nierenfunktion sowie eine weitere Studie zum akuten Nierenversagen nach MitraClip®-Implantation (Abbott, Chicago, IL, USA) geben wichtige Hinweise zu zukünftigen Handlungsempfehlungen. Der optimale Zeitpunkt der Einleitung einer Nierenersatztherapie bei Patienten mit akuter Nierenschädigung in der Intensivmedizin wurde in 2 randomisierten Studien untersucht, die entsprechend diskutiert werden.

## Abstract (en)

Patients with chronic kidney diseases show an increased cardiovascular morbidity and mortality. Last year a number of important studies on heart-kidney interaction were published, which are summarized and discussed in this article. In the DAPA-CKD study and the SCORED study two different sodium-glucose linked transporter 2 (SGLT2) inhibitors (dapagliflozin and sotagliflozin) were found to improve the prognosis of patients with chronic kidney diseases with and without diabetes. The results of the randomized study on the new mineralocorticoid receptor antagonist finerenon (FIDELIO-DKD) also provided a very promising novel treatment approach for patients with diabetic nephropathy. The published data of the ISCHEMIA-CKD study in patients with coronary heart disease and investigations on the influence of transcatheter aortic valve implantation (TAVI) on renal function as well as another study on acute kidney failure after MitraClip® (Abbott, Chicago, IL, USA) implantation provide important indications for future treatment recommendations. The optimal timing of the initiation of kidney replacement therapy in patients with acute kidney damage in intensive care medicine was investigated in two randomized studies, which are correspondingly discussed.

## Einleitung

Patienten, die an einer chronischen Herzinsuffizienz leiden, sind besonders häufig von einer chronischen Nierenfunktionseinschränkung („chronic kidney disease“, CKD) betroffen [1]. Ohne adäquate medikamentöse Behandlung, welche insbesondere die Therapie begleitender Risikofaktoren wie Diabetes mellitus und arterielle Hypertonie beinhaltet, ist diese vulnerable Patientengruppe von einer deutlich erhöhten kardiovaskulären Sterblichkeit betroffen [2].

Auch für Patienten mit höhergradigen Herzklappenvitien [3], wie die hochgradige Aortenklappenstenose oder die hochgradige Mitralklappeninsuffizienz, sowie für Patienten mit koronarer Herzkrankheit (KHK; [4]) ist die zugrunde liegende Nierenfunktion von Relevanz. Insbesondere dann, wenn eine Intervention – sowohl durch interventionelle als auch chirurgische Verfahren – vorgesehen ist. Entsprechende Handlungsempfehlungen sind daher wichtig. Dies gilt auch für die Einleitung von Nierenersatzverfahren in der Intensivmedizin.

## Medikamentöse Therapieoptionen

Bisher konnte in randomisierten, kontrollierten Studien nur die Therapie mittels ACE („angiotensin-converting enzyme“)-Hemmer bzw. AT1(Angiotensin-II-Rezeptor Subtyp 1)-Blocker (Angiotensinrezeptorblocker, ARB) bei dieser Patientengruppe eine Prognoseverbesserung zeigen. Mit den vor wenigen Jahren eigentlich als orale Antidiabetika zugelassenen SGLT2(„sodium-glucose linked transporter 2“)-Inhibitoren steht nun eine wichtige neue Therapieoption für herz- und nierenkranke Patienten zur Verfügung.

Nach den positiven Ergebnissen der Studienprogramme zu Empagliflozin (EMPA-REG-OUTCOME [5, 6]/EMPEROR-Reduced [7]), Canagliflozin (CANVAS/CANVAS‑R [8]/CREDENCE [9]) und Dapagliflozin (DECLARE-TIMI-58 [10]/DAPA-HF [11]), welche alle eine Reduktion der Rate an herzinsuffizienzbedingter Hospitalisierung sowie eine Reduktion des renalen Endpunkts bei Patienten mit Diabetes mellitus zeigten (Tab. 1), wurden in DAPA-CKD nun erstmals auch chronisch nierenkranke Patienten mit und ohne Diabetes mellitus untersucht [12].

**Table 1:** 

Studie	Kohorte	*n*	Follow-up*	Primärer Endpunkt	Ergebnisse
*Empagliflozin*					
EMPA-REG OUTCOME	DM Typ II + hohes KV-Risiko (+ eGFR ≥ 30 ml/min/1,73 m^2^)	7020	3,1 Jahre	1 EP: 3 MACE	HR: 0,86 (95 %-KI: 0,74–0,99)
EMPA-KIDNEY (NCT03594110)	CKD (eGFR: 20–45 ml/min/1,73 m^2^ oder 45–90 ml/min/1,73 m^2^ + UACR ≥ 200 mg/g)	> 6000	≥ 1 Jahr	ESKD/renaler Tod/eGFR-Abfall ≥ 40 % oder KV-Tod	Erwartet Oktober 2022
EMPEROR-Reduced	HFrEF (LVEF < 40 %)	3730	16 Monate	KV-Tod + HI	HR: 0,75 (95 %-KI: 0,65–0,86)
EMPEROR-Preserved (NCT03057951)	HFpEF (LVEF > 40 %)	> 5988	≥ 1 Jahr	KV-Tod + HI	Erwartet März 2021
*Dapagliflozin*					
DECLARE-TIMI 58	DM Typ II + hohes KV-Risiko	17.160	4,2 Jahre	MACE + KV-Tod oder HI	HR: 0,93 (95 %-KI: 0,84–1,03) HR: 0,83 (95 %-KI: 0,73–0,95)
DAPA-CKD	CKD (eGFR: 25–75 ml/min/1,73 m^2^ + UACR: 200–5000 mg/g)	4304	2,4 Jahre	ESKD/renaler Tod/eGFR-Abfall ≥ 50 % oder KV-Tod	HR: 0,61 (95 %-KI: 0,51–0,72)
DAPA-HF	HFrEF (LVEF < 40 %)	4744	18,2 Monate	KV-Tod oder HI	HR: 0,74 (95 %-KI: 0,65–0,85)
DELIVER (NCT03619213)	HFpEF (LVEF > 40 %)	> 6100	≥ 1 Jahr	KV-Tod oder HI	Erwartet November 2021
*Canagliflozin*					
CANVAS/CANVAS‑R	DM Typ II + hohes KV-Risiko	10.142	188,2 Monate	3 MACE	HR: 0,86 (95 %-KI: 0,75–0,97)
CREDENCE	DM Typ II + CKD (eGFR: 30–90 ml/min/1,73 m^2^ + UACR: 300–5000 mg/g)	4401	2,62 Jahre	ESKD/renaler Tod/Verdopplung SCr oder KV-Tod	HR: 0,70 (95 %-KI: 0,59–0,82)
*Sotagliflozin*					
SCORED	DM Typ II + CKD (eGFR: 25–60 ml/min/1,73 m^2^) + hohes KV-Risiko	10.584	16 Monate	KV-Tod + HI	HR: 0,74 (95 %-KI: 0,63–0,88)
SOLOIST WHF	DM Typ II + WHF	1222	9 Monate	KV-Tod + HI	HR: 0,67 (95 %-KI: 0,52–0,85)
*Ertugliflozin*					
VERTIS CV	DM Typ II + prävalente CVD	8246	3,5 Jahre	3 MACE	HR: 0,97 (95 %-KI: 0,85–1,11)

*DM* Diabetes mellitus; *eGFR* „estimated glomerular filtration rate“ (geschätzte glomeruläre Filtrationsrate); *KV* kardiovaskulär; *EP* Endpunkt; *UACR* „urine albumin-to-creatinine ratio“ (Albumin-Kreatinin-Quotient im Urin); *HR* Hazard Ratio; *KI* Konfidenzintervall; *HFrEF* „heart failure with reduced ejection fraction“ (Herzinsuffizienz mit eingeschränkter systolischer Ejektionsfraktion); *CKD* „chronic kidney disease“ (chronische Nierenerkrankung); *HFpEF* „heart failure with preserved ejection fraction“ (Herzinsuffizienz mit erhaltener systolischer Ejektionsfraktion); *LVEF *„left ventricular ejection fraction“ (linksventrikuläre Auswurffraktion); *HI* herzinsuffizienzbedingte Hospitalisierung; *MACE* „major cardiovascular events“ (schwere kardiovaskuläre Ereignisse: kardiovaskulärer Tod, akuter Myokardinfarkt, Apoplex); *ESKD* „end-stage kidney disease“ (terminale Niereninsuffizienz); *SCr* Serumkreatinin; *CVD* „cardiovascular diesease“ (kardiovaskuläre Erkrankung); *WHF* „recently hospitalization for worsening of heart failure“ (rezente Hospitalisierung aufgrund einer sich verschlechternden Herzinsuffizienz); *Nachbeobachtungszeitraum, als Median oder Mittelwert

### SGLT2-Inhibiton – DAPA-CKD

In die DAPA-CKD-Studie wurden 4304 chronisch nierenkranke Patienten der KDIGO(Kidney Disease: Improving Global Outcomes)-GFR-Stadien G2 bis G4 (eGFR [„estimated glomerular filtration rate“]: 25–75 ml/min/1,73 m^2^; Median: 43 ml/min/1,73 m^2^) sowie einem Albumin-Kreatinin-Quotienten im Urin (UACR) zwischen 200 und 5000 mg/g (median: 950 mg/g) eingeschlossen [12]. Insgesamt 67 % der Patienten wiesen einen Diabetes mellitus Typ II auf. Das mittlere Alter lag im Schnitt bei 62 Jahren, und 33 % der Patienten waren Frauen. Fast alle Studienteilnehmer wurden leitliniengerecht mit einem ACE-Hemmer oder einem ARB behandelt.

Es erfolgte eine Randomisierung in einen Behandlungsarm, der mit dem SGLT2-Inhibitor Dapagliflozin (10 mg/Tag) behandelt wurde, bzw. in einen Placeboarm. Der zusammengesetzte primäre Endpunkt wurde definiert als ein 50%iger Abfall der eGFR bzw. das Auftreten einer terminalen Niereninsuffizienz und/oder das Eintreten eines renalen oder kardiovaskulären Todes. Nach einem durchschnittlichen Nachbeobachtungszeitraum von 2,4 Jahren wurde die Studie aufgrund eines Behandlungsvorteils für die Dapagliflozingruppe vorzeitig abgebrochen.

Der primäre Endpunkt wurde von 197 Patienten der Dapagliflozingruppe und 312 Patienten der Placebogruppe bis zum Zeitpunkt des Studienabbruchs erreicht. Dies entspricht einer Reduktion des relativen Risikos (RR) um 39 % (Hazard Ratio [HR]: 0,61; 95 %-Konfidenzintervall [KI]: 0,51–0,72; *p* < 0,001). Auch im Hinblick auf die sekundären Endpunkte zeigte sich eine Behandlung mittels Dapagliflozin gegenüber Placebo überlegen: So wurde für den renalen Endpunkt (50%iger Abfall der eGFR, terminale Niereninsuffizienz oder renaler Tod) das RR um 44 % (HR: 0,56; 95 %-KI: 0,45–0,68; *p* < 0,001) reduziert. Für den kombinierten Endpunkt aus kardiovaskulärem Tod oder Hospitalisierung aufgrund akut dekompensierter Herzinsuffizienz reduzierte sich das RR um 29 % (HR: 0,71; 95 %-KI: 0,55–0,92; *p* = 0,009). Weiterhin lag die RR-Reduktion für die Gesamtsterblichkeit bei 31 % (HR: 0,69; 95 %-KI: 0,53–0,88; *p* = 0,004).

Die Prognoseverbesserung zeigte sich für Patienten sowohl mit als auch ohne Diabetes, und die Effekte traten – bei insgesamt guter Verträglichkeit – unabhängig von der Nierenfunktion und der Schwere der Albuminurie auf [12].

In DAPA-CKD konnte erstmals auch für chronisch nierenkranke Patienten mit und ohne Diabetes mellitus Typ II eine Prognoseverbesserung unter der Einnahme von Dapagliflozin aufgezeigt werden.

Weiterhin wurden im vergangenen Jahr auch Daten zu Sotagliflozin zur Behandlung von Patienten mit Typ-2-Diabetes mit CKD (mit und ohne Albuminurie) veröffentlicht. Im Gegensatz zu den zuvor genannten SGLT2-Inhibitoren hemmt Sotagliflozin auch SGLT1 im Darm. Das Studienprogramm wurde nach 16 Monaten aufgrund von Finanzierungsproblemen vorzeitig beendet. Zwar kam es zu einer RR-Reduktion von 26 % (HR: 0,74; 95 %-KI: 0,63–0,88; *p* < 0,001) unter der Behandlung mit Sotagliflozin hinsichtlich des während des Studienverlaufs neu definierten primären Endpunkts (kardiovaskulärer Tod oder herzinsuffizienzbedingte Hospitalisierung oder notfallmäßige Vorstellung aufgrund Verschlechterung einer bekannten Herzinsuffizienz), allerdings traten auch vermehrte Nebenwirkungen (Diarrhöen, diabetische Ketoazidose, genitale Pilzinfektionen und Volumenmangel) in dieser Behandlungsgruppe auf [13].

### Mineralokortikoidrezeptorantagonist Finerenon – FIDELIO-DKD

Die Inhibition des Renin-Angiotensin-Aldosteron-Systems (RAAS) zur Reduktion von Inflammation und Fibrosierung ist von erheblicher therapeutischer Relevanz. Bisher konnte in klinischen Studien eine Erweiterung der medikamentösen Behandlung (neben ACE-Hemmern oder ARB) um einen Mineralokortikoidrezeptorantagonisten wie Spironolacton oder Eplerenon keinen weiteren Prognosevorteil für Patienten mit CKD und Diabetes mellitus zeigen [14]. Mit Finerenon ist ein neuartiger, nichtsteroidaler, selektiver Mineralokortikoidrezeptorantagonist mit höherer Rezeptorspezifität als Spironolacton und Eplerenon erstmals in einer randomisierten, doppelblinden, placebokontrollierten, multizentrischen und ereignisgesteuerten Phase-III-Studie (FIDELIO-DKD) untersucht worden [15]. Finerenon konnte als erster Vertreter dieser Substanzklasse einen Nutzen im Hinblick auf kardiovaskuläre und renale Ereignisse bei Patienten mit diabetischer Nephropathie (eGFR: 25–59 ml/min/1,73 m^2^ + UACR: 30–299 mg/g + diabetische Retinopathie oder eGFR: 25–74 ml/min/1,73 m^2^ +UACR: 300–5000 mg/g) nachweisen.

Insgesamt wurden 5734 Teilnehmer eingeschlossen, auf Finerenon 10 mg bzw. 20 mg oder Placebo randomisiert und im Median über 2,6 Jahre nachverfolgt. Alle Teilnehmer wurden bereits vor Studieneinschluss leitliniengerecht mit einer maximal tolerierten Dosierung eines ACE-Hemmers oder eines ARB behandelt. Der primäre Endpunkt setzte sich aus terminaler Niereninsuffizienz (Dialysepflichtigkeit, Nierentransplantation oder Abfall der eGFR < 15 ml/min/1,73 m^2^), anhaltender Nierenfunktionsverschlechterung (Abfall der eGFR um 40 % über ≥ 4 Wochen) und renal bedingtem Tod zusammen [15].

**Figure 1:**
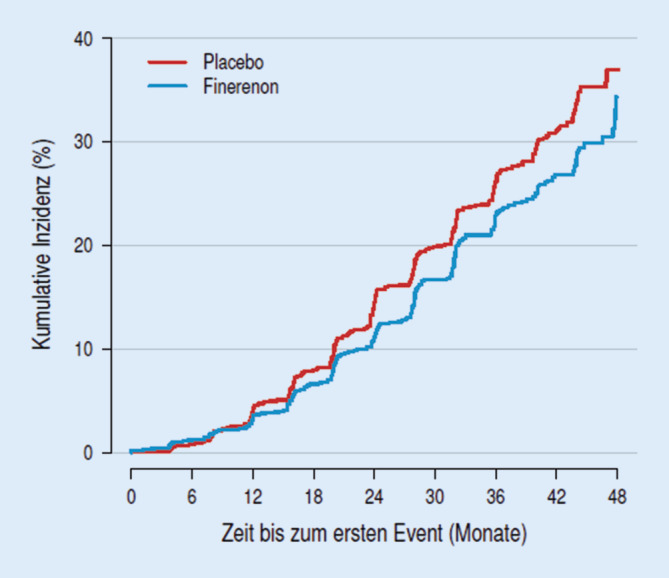

Im Behandlungsarm mit Finerenon kam es im Hinblick auf diesen primären Endpunkt zu einer RR-Reduktion von 18 % gegenüber der Placebogruppe (HR: 0,82; 95 %-KI: 0,73–0,93; *p* < 0,001; Abb. 1). Dies galt auch für den sekundären Endpunkt, definiert als Kombination aus kardiovaskulärem Tod, akutem Myokardinfarkt, Apoplex und herzinsuffizienzbedingter Hospitalisierung. Hier zeigte sich eine RR-Reduktion um 14 % für die Finerenonbehandlungsgruppe (HR: 0,86; 95 %-KI: 0,75–0,99; *p* = 0,03). Hinsichtlich unerwünschter Ereignisse zeigten sich im Finerenonbehandlungsarm häufiger Hyperkaliämien (18,3 % vs. 9,0 %), wobei schwerwiegende hyperkaliämiebedingte Ereignisse insgesamt selten auftraten (1,6 % vs. 0,4 %). Es kam zu keinem hyperkaliämiebedingten Todesfall. Insgesamt 2,3 % der mit Finerenon behandelten Teilnehmer im Vergleich zu 0,9 % der Placebogruppe mussten die Teilnahme aufgrund einer Hyperkaliämie abbrechen. Der blutdrucksenkende Effekt des mittleren systolischen Blutdrucks war mit 2–3 mm Hg unter Finerenontherapie moderat [15].

Aufgrund der Ergebnisse ausFIDELIO-DKD gewährte die US-amerikanische Zulassungsbehörde(Food and Drug Administration [FDA]) den Status der vorrangigen Prüfung („priority review“), sodass bereits im Juni 2021 mit einer Zulassungsentscheidung zu rechnen ist. Auch in der Europäischen Union (EU) wurde aufgrund dieser Daten die Zulassung für Finerenon beantragt.

Aktuell (Februar 2021) werden auch die ersten Vorabergebnisse aus FIGARO-DKD (NCT02545049) erwartet, welche in einem ebensolchen Kollektiv das kardiovaskuläre Outcome unter Finerenon im Vergleich zu Placebo überprüft.

Mit Finerenon konnte für eine weitere Substanzklasse eine Prognoseverbesserung für Diabetespatienten mit CKD sowohl für renale als auch für kardiovaskuläre Ereignisse nachgewiesen werden, indem es Inflammation und Fibrose entgegenwirkt.

## Neue Blutdruckleitlinien der KDIGO

Die KDIGO hat im Jahr 2020 ihre aktualisierten Leitlinien zur Hypertonie bei CKD veröffentlicht [16]. Dabei wird erstmals eine Senkung des systolischen Blutdrucks auf Werte unterhalb von 120 mm Hg (standardisierter Praxisblutdruck) für nichtdialysepflichtige, chronisch nierenkranke Patienten empfohlen. Die neuen Zielwerte basieren im Wesentlichen auf den Ergebnissen der SPRINT- [17, 18] und der ACCORD-Studie [19] und liegen unterhalb der Zielwertempfehlungen von US-amerikanischen (American College of Cardiology [ACC]/American Heart Association [AHA]; [20]) und europäischen Leitlinien (European Society of Cardiology [ESC]/European Society of Hypertension [ESH]; [21]; Tab. 2; [22]). Zielwerte für diastolische Blutdruckwerte wurden bewusst nicht festgelegt, auch verzichtete man im Gegensatz zu den vorherigen Leitlinien – bei jetzt insgesamt niedrigeren systolischen Zielblutdruckwerten – auf die Unterscheidung zwischen dem Vorliegen einer Albuminurie oder keiner Albuminurie. Die Zielwerte für Nierentransplantierte liegen in den neuen Leitlinien unverändert bei weniger als 130/80 mm Hg.

**Table 2:** 

	CKD (ohne Dialysepflichtigkeit)	Nierentransplantierte
KDIGO	< 120 mm Hg^a^	≤ 130/80 mm Hg
ACC/AHA	< 130/80 mm Hg	n. a.
ESC/ESH	< 140/90 mm Hg (in Richtung 130/80 mm Hg)	n. a.

*ACC* American College of Cardiology, *AHA* American Heart Association, *ESC* European Society of Cardiology, *ESH* European Society of Hypertension, *KDIGO* Kidney Disease: Improving Global Outcomes; *CKD* „chronic kidney disease“ (chronische Nierenerkrankung), *n.* *a.* nicht angegeben

^a^diastolische Zielwerte wurden bewusst nicht definiert

Zur Hypertoniebehandlung werden begleitende Lebensstilmaßnahmen empfohlen, wie die Reduktion des Salzkonsums (absolute Einnahme von Natriumchlorid) auf weniger als 5 g/Tag und ausreichende körperliche Bewegung von mindestens 150 min/Woche. Die medikamentöse Behandlung mittels ACE-Hemmer oder ARB wird – unter regelmäßiger Kontrolle der Serumkreatinin- und Serumkaliumwerte – für Patienten sowohl mit als auch ohne Diabetes in den KDIGO-GFR-Stadien G1–G4, A2–A3 sowie für Diabetespatienten ohne Albuminurie und einer eGFR von weniger als 60 ml/min/1,73 m^2^ unter Titration bis zur maximalen Dosis empfohlen [16].

Für Patienten mit CKD und therapieresistenter Hypertonie sollte eine Kombination mit bisher zugelassenen Mineralokortikoidrezeptorantagonisten wie Spironolacton oder Eplerenon nur dann erfolgen, wenn sie in der Vergangenheit keine Hyperkaliämie zeigten und eine eGFR von mehr als 45 ml/min/1,73 m^2^ aufweisen. Für chronisch nierenkranke Patienten sämtlicher Stadien mit und ohne Diabetes ist die Kombination von ACE-Hemmern bzw. ARB und direkten Renininhibitoren aufgrund des erhöhten Risikos für das Auftreten einer Hyperkaliämie oder eines akuten Nierenversagens zu vermeiden [16].

Die neuen KDIGO-Leitlinien empfehlen einen systolischen Zielblutdruckwert für chronisch nierenkranke, nichtdialysepflichtige Patienten von weniger als 120 mm Hg; ein diastolischer Zielblutdruckwert wurde nicht festgelegt.

## ACE-Hemmer/ARB beenden oder fortführen bei Nierenfunktionseinschränkung?

Unter Therapie mit ACE-Hemmern oder ARB kann es zu einer Nierenfunktionsverschlechterung mit begleitender Hyperkaliämie kommen. Obwohl die Datenlage für ACE-Hemmer und ARB bezüglich der kardiovaskulären und renalen Prognose durchweg vorteilhaft ist, bedingen ein Abfall der eGFR und/oder das Auftreten einer Hyperkaliämie – meist verursacht durch die Hemmung der Produktion von Angiotensin II und der Abnahme der Aldosteronsekretion – klinisch oftmals ein Pausieren oder Absetzen dieser Substanzen. Eine Studie aus Pennsylvania, USA, untersuchte nun anhand der daten von 3909 Patienten, ob das Absetzen der ACE-Hemmer oder ARB innerhalb von 6 Monaten nach eGFR-Abfall prognostische Auswirkungen hinsichtlich kardiovaskulärer Ereignisse (Tod, akuter Myokardinfarkt, Koronarinterventionen oder Bypassoperation), der Sterblichkeitsrate oder des Auftretens eines akuten Nierenversagens hatte [23]. Als Schwellenwert wurde eine eGFR von 30 ml/min/1,73 m^2^ definiert. Kam es zu einem Abfall der eGFR auf Werte unterhalb von 30 ml/min/1,73 m^2^, wurde bei 1235 Patienten die Einnahme eines ACE-Hemmers bzw. eines ARB beendet, bei 2674 Patienten wurde die Einnahme trotz eGFR-Abfall fortgeführt [23].

**Figure 2:**
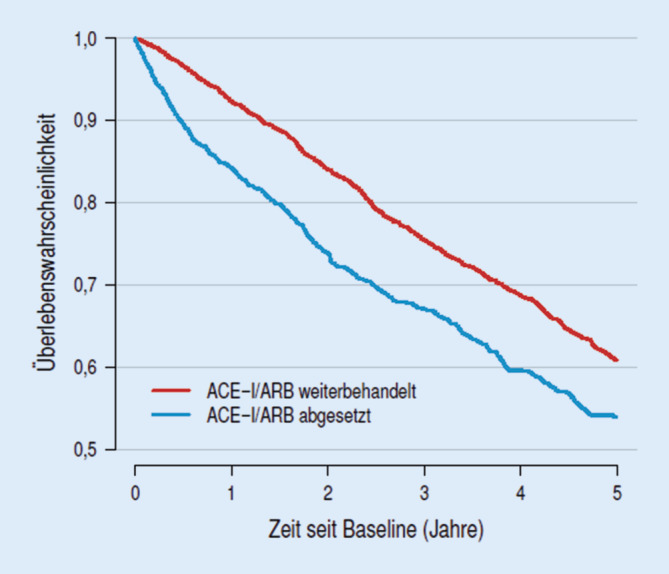

Im medianen Nachbeobachtungszeitraum von 2,9 Jahren war das Absetzen der ACE-Hemmer- bzw. der ARB-Therapie mit einem höheren Sterblichkeitsrisiko assoziiert (HR: 1,39; 95 %-KI: 1,20–1,60; Abb. 2). Dies galt auch für das Auftreten von kardiovaskulären Komplikationen. Patienten, bei denen die RAAS-Inhibition aufgrund einer Nierenfunktionsverschlechterung beendet wurde, hatten ein relativ höheres Risiko für das Auftreten von schweren kardiovaskulären Ereignissen („major adverse cardiac events“ [MACE]; HR: 1,37; 95 %-KI: 1,20–1,56). Im Hinblick auf das Auftreten einer terminalen Nierenfunktionseinschränkung gab es keine Unterschiede. Bei Patienten, bei denen die ACE-Hemmer- bzw. die ARB-Therapie beendet wurde, kam es weniger häufig zu Hyperkaliämien (HR: 0,65; 95 %-KI: 0,54–0,79; [23]). Einschränkend muss gesagt werden, dass es sich bei der vorliegenden Studie um eine reine Beobachtungsstudie handelt.

Unter Fortführung der Behandlung mit ACE-Hemmer oder ARB kam es trotz eGFR-Abfalls auf Werte unter 30 ml/min/1,73 m^2^ nicht zu erhöhten Raten einer terminalen Niereninsuffizienz. Das Risiko, zu versterben oder ein kardiovaskuläres Ereignis zu erleiden, war unter fortgeführter Therapie niedriger. Allerdings nahm die Rate an Hyperkaliämien zu.

## Interventionelle Klappentherapie und Niereninsuffizienz (TAVI und Mitraclip® [Abbott, Chicago, IL, USA])

Patienten mit CKD sind besonders häufig bereits in frühen Stadien von Herzklappenvitien wie Aortenklappenstenose oder Mitralklappeninsuffizienz betroffen [3]. Die Mehrzahl der Patienten mit seniler kalzifizierter Aortenklappenstenose, die mit einem interventionellen Aortenklappenersatz („transcatheter aortic valve implantation“, TAVI) behandelt werden, weist auch eine begleitende chronische Nierenfunktionseinschränkung auf. Eine rezent veröffentlichte Post-hoc-Analyse der PARTNER-Studien (PARTNER 1, 2, S3) an 5190 Patienten konnte dabei zeigen, dass die vorliegende Nierenfunktionseinschränkung auch die Prognose nach der Intervention beeinflusst [24, 25]. Insgesamt 91 % der Teilnehmer hatten zum Untersuchungsbeginn eine eGFR von weniger als 90 ml/min/1,73 m^2^ (KDIGO-GFR-Stadium < G2); periinterventionell wurden durchschnittlich 100 ml Kontrastmittel appliziert. Patienten mit geringerer Nierenfunktion hatten im Vergleich zu denen mit höherer Nierenfunktion eine höhere Sterblichkeit im postinterventionellen Verlauf. Die Nierenfunktion stabilisierte oder verbesserte sich nach der TAVI bei 89 % der untersuchten Patienten. Das Risiko, eine dialysepflichtige Nierenfunktionseinschränkung zu erleiden, lag bei nur 0,035 % [25].

Als mögliche zugrunde liegende Mechanismen, die eine Verbesserung der Nierenfunktion erklären könnten, werden die Erhöhung des „cardiac output“, die Reduktion intrakardialer Drücke und der Vorlast sowie eine Reduktion der Aktivität des Sympathikus- sowie des RAAS-Systems diskutiert [25].

Diese Daten unterstreichen die Bedeutung der Aortenklappenstenose im Rahmen des kardiorenalen Syndroms. Eine Nierenfunktionseinschränkung sollte nicht als Kontraindikation für eine TAVI verstanden werden.

Auch die hochgradige sekundäre Mitralklappeninsuffizienz bei Herzinsuffizienz ist eine relevante Komorbidität der CKD. Die interventionelle Edge-to-edge-Reparatur mittels MitraClip®-Implantation hat sich bei ausgewählten Patienten als sichere und effektive Methode etabliert. Eine rezent veröffentlichte Studie untersuchte an 721 Patienten mit hochgradiger Mitralklappeninsuffizienz das Risiko des Auftretens eines akuten Nierenversagens (Serumkreatininanstieg > 0,3 mg/dl oder > 50 % gegenüber dem Ausgangsserumkreatinin oder Einleitung einer Hämodialyse während der Hospitalisierung) nach MitraClip®-Implantation [26]. Die Teilnehmer waren im Schnitt 72 Jahre alt und hatten eine mittlere eGFR von 44 ml/min/1,73 m^2^, bzw. 75 % der Patienten wiesen definitionsgemäß eine CKD auf. Bei 15 % der Patienten trat postinterventionell ein akutes Nierenversagen auf, obwohl bei der MitraClip®-Implantation in der Regel kein Kontrastmittel verwendet wird. Patienten, bei denen ein akutes Nierenversagen auftrat, hatten insgesamt eine schlechtere Prognose. Risikofaktoren für das Auftreten eines akuten Nierenversagens waren eine Anämie, die Gabe von nephrotoxischen Substanzen, eine Vollnarkose, transiente Hypotonien, Blutungsereignisse und eine nicht erfolgreiche Prozedur. Neben einer höheren Rate an Blutungskomplikationen (19,4 % vs. 2,2 %; *p* < 0,001) kam es zu einer höheren Sterblichkeit nach 2 Jahren (38,6 % vs. 17,8 %; *p* < 0,001) und einer höheren Rate an MACE (58,5 % vs. 22,3 %; *p* < 0,001). Weiterhin nahm bei diesen Patienten die Nierenfunktion in den folgenden 12 Monaten signifikant um 20 % ab (eGFR: von 41,8 auf 33,4 ml/min/1,73 m^2^; *p* < 0,001), wohingegen sich die Nierenfunktion bei Patienten ohne postinterventionelles akutes Nierenversagen um 3 % stabilisierte [26].

Ein akutes Nierenversagen nach MitraClip®-Implantation trat bei 1 von 6 Patienten auf und war mit perioperativen Komplikationen assoziiert. Im Gegensatz zu Patienten ohne hatten Patienten mit akutem Nierenversagen eine insgesamt schlechtere postinterventionelle Prognose.

## Zeitpunkt der Nierenersatztherapie bei Patienten mit akuter Nierenschädigung

Die akute Nierenschädigung ist bei kritisch kranken Patienten häufig zu finden und mit einer hohen Sterblichkeit und schwerwiegenden Komplikationen assoziiert [27]. Treten bei fortgeschrittener akuter Nierenschädigung lebensbedrohliche Komplikationen, beispielsweise Stoffwechselstörungen in Form von schwerer metabolischer Azidose, Hyperkaliämie oder Hypervolämie, auf, ist zur Korrektur dieser Störungen das Einleiten einer Nierenersatztherapie indiziert. Zahlreiche Studien beschäftigten sich mit der Hypothese, ob eine frühzeitige (bzw. vorzeitige) Einleitung einer Nierenersatztherapie im Vergleich zur standardmäßigen Indikationsstellung mit einer geringeren Sterblichkeit assoziiert ist [28]. In einer systematischen Metaanalyse von individuellen Patientendaten ausgewählter randomisierter Studien wurden die Effekte eines frühen (2–8 h) oder verzögerten Beginns (25–57 h) einer Nierenersatztherapie hinsichtlich der 28-Tages-Sterblichkeit (primärer Endpunkt) untersucht [28]. Eingeschlossen wurden 2143 kritisch kranke Patienten aus 10 randomisierten Studien mit schwerer akuter Nierenschädigung und KDIGO/AKI(„acute kidney injury“)*-*Stadium 2–3 oder einem SOFA(„sepsis-related organ failure assessment“)-Score von 3 oder mehr, bezogen auf die Nierenfunktion. Von insgesamt 1664 untersuchten Patienten, zu denen es individuelle Daten in Bezug auf diesen Endpunkt gab, erreichten 366 (44 %) in der „verzögerten“ und 355 (43 %) in der „frühzeitigen“ Gruppe den primären Endpunkt (RR: 1,01; 95 % KI 0,91–1,13; *p* = 0,80). Hinsichtlich schwerer Nebenwirkungen (Hyperkaliämien, Blutungen oder Herzrhythmusstörungen) zeigten sich keine signifikanten Unterschiede zwischen den Gruppen. Auch in verschiedenen Subgruppen hatte das Vorliegen einer CKD oder ein unterschiedlich hoher SOFA-Ausgangsscore keinen Einfluss auf den Behandlungseffekt. Insgesamt 42 % der Patienten in der „verzögerten“ Gruppe erhielten im Untersuchungszeitraum keine Nierenersatztherapie. Anhand dieser metaanalytischen Ergebnisse lässt sich schlussfolgern, dass kein signifikanter Unterschied in der 28-Tages-Sterblichkeit bei Patienten mit akuter Nierenschädigung hinsichtlich des Startzeitpunkts einer Nierenersatztherapie vorliegt und keine Vorteile aus einem vorzeitigen Beginn dieser Therapie resultieren [28]. Ohne Vorliegen einer Dialyseindikation sollte eine Nierenersatztherapie bei akuter Nierenschädigung nicht mehr vorzeitig durchgeführt werden. Es konnte zudem gezeigt werden, dass ein früh-/vorzeitiger Beginn einer Nierenersatztherapie nicht nur mit einer erhöhten Komplikationsrate, sondern auch in 35–49 % der Fälle mit einer „unnötigen“ Nierenersatztherapie assoziiert war [29, 30].

Die kürzlich erschienene STARRT-AKI-Studie verglich ebenfalls unterschiedliche Zeitpunkte der Initiierung einer Nierenersatztherapie und bestätigte die Ergebnisse der oben diskutierten Metaanalyse [31]. Insgesamt 2927 Patienten mit akuter Nierenschädigung (KDIGO-AKI-Stadium 2–3) erhielten nach erfolgter Randomisierung in der „frühzeitigen“ Gruppe im Median nach 6,1 h eine Nierenersatztherapie, wohingegen die „Standardtherapie“-Gruppe bis zum Erfüllen konservativer Indikationskriterien (Serumkalium > 6,0 mmol/l; pH < 7,20; Serumbikarbonat < 12 mmol/l, lebensbedrohliche Volumenüberladung) ohne Nierenersatztherapie behandelt wurde. Die Kriterien zur Einleitung einer Nierenersatztherapie wurden von 61,8 % der Patienten im Verlauf von im Median 31,1 h erfüllt. Den primären Endpunkt erreichten 643 (43,9 %) Patienten in der „frühzeitigen“ und 639 (43,7 %) in der „Standardstrategie“-Gruppe. Es zeigte sich kein signifikanter Unterschied zwischen beiden Gruppen hinsichtlich der 90-Tages-Sterblichkeit (RR: 1,00; 95 %-KI: 0,93–1,09). Nennenswert ist allerdings, dass in der „frühzeitigen“ Gruppe die dauerhafte Dialysepflichtigkeit (RR: 1,74; 95 %-KI: 1,24–2,43) sowie die Anzahl der Rehospitalisierungen nach 90 Tagen signifikant höher waren (RR: 1,23; 95 %-KI: 1,02–1,49). Zusätzlich traten unerwünschte Ereignisse, am häufigsten in Form von Blutdruckabfällen oder Hypophosphatämien, in der „frühzeitigen“ Gruppe signifikant häufiger auf (RR: 1,40; 95 %-KI: 1,21–1,62; *p* < 0,001; [31]).

Ein vorzeitiger Einsatz eines Nierenersatzverfahrens im Vergleich zur Standardtherapie ist nicht mit einer Prognoseverbesserung assoziiert, erhöht aber die Komplikationsrate.

## ISCHEMIA-CKD

Bei Patienten mit CKD sind kardiovaskuläre Erkrankungen die häufigste Todesursache. Die KHK kann mittels medikamentöser, konservativer bzw. Revaskularisationstherapien via perkutane Koronarintervention bzw. Bypassoperation behandelt werden. Invasive Behandlungsansätze in Form einer Koronarangiographie oder einer Revaskularisationstherapie bei niereninsuffizienten Patienten können mit Komplikationen verbunden sein. Es stellt sich daher die Frage, ob dieses erhöhte Risiko im Vergleich zur konservativen medikamentösen Therapie mit einem längerfristig geringeren Auftreten kardiovaskulärer Ereignisse bei Patienten mit Niereninsuffizienz und chronischem Koronarsyndrom (CCS) einhergeht. Dieser Frage widmete sich die ISCHEMIA-CKD-Studie, in welche mehr als 750 Patienten mit einer schweren Nierenfunktionseinschränkung (eGFR < 30 ml/min/1,73 m^2^ oder bestehende Dialysepflichtigkeit) und gleichzeitig vorliegendem CCS mit stressinduzierter moderater oder schwerer Myokardischämie eingeschlossen wurden [32]. Nach erfolgter Randomisierung erhielt die „initial-invasive“ Gruppe innerhalb der folgenden 30 Tage eine Koronarangiographie zusätzlich zur medikamentösen Standardtherapie, welche bei vorhandener Indikation ggf. durch eine perkutane Koronarintervention oder eine koronararterielle Bypassoperation ergänzt wurde. Die „konservative“ Gruppe erhielt eine aus Lebensstilmodifikation und medikamentöser Standardtherapie bestehende Behandlung, welche jedoch bei Auftreten von Komplikationen oder Unwirksamkeit dieser Behandlungsform auf eine invasive Therapie umgestellt wurde. Den kombinierten primären Endpunkt, bestehend aus Tod und nichttödlichem Myokardinfarkt, erreichten nach einer medianen Beobachtungszeit von 2,2 Jahren in der „initial-invasiven“ Gruppe 123 Patienten und in der „konservativen“ Gruppe 129 Patienten mit einer geschätzten 3‑Jahres-Inzidenz von 36,4 % und 36,7 % (HR: 1,01; 95 %-KI: 0,79–1,29). Es zeigte sich somit kein signifikanter Unterschied zwischen den Gruppen hinsichtlich des Auftretens des primären Endpunkts. Zusätzlich zeigte sich im Gruppenvergleich ebenfalls kein signifikanter Unterschied bezüglich des kombinierten sekundären Endpunkts, welcher ergänzend die Hospitalisierung im Rahmen einer instabilen Angina pectoris, einer Herzinsuffizienz oder eines wiederbelebten Herzkreislaufstillstands einschloss. Allerdings hatten Patienten aus der „initial-invasiven“ Gruppe eine signifikant höhere Inzidenz für Schlaganfälle (HR: 3,76; 95 %-KI: 1,52–9,32; [32]).

Wichtig ist es, darauf hinzuweisen, dass Patienten mit Herzinsuffizienz und Hauptstammstenose nicht in die Untersuchung eingeschlossen worden sind. Ferner fehlen bislang Daten, die den Einfluss der prozeduralen FFR(fraktionelle Flussreserve)-Messung auf das Studienergebnis untersuchten.

Eine initial-invasive Therapie bei Patienten mit schwerer CKD und gleichzeitig bestehendem CCS verringerte in ISCHEMIA-CKD nicht die Mortalität oder das Auftreten von nichttödlichem Myokardinfarkt im Vergleich zur konservativen Strategie.

## References

[1] Hebert K, Dias A, Delgado MC (2010). Epidemiology and survival of the five stages of chronic kidney disease in a systolic heart failure population. Eur J Heart Fail.

[2] Hillege HL, Girbes ARJ, De Kam PJ (2000). Renal function, neurohormonal activation, and survival in patients with chronic heart failure. Circulation.

[3] Ewen S, Mahfoud F, Lauder L (2020). Valvular heart disease in patients with chronic kidney disease. Internist.

[4] Lauder L, Ewen S, Emrich IE (2019). Cardiovascular pharmacotherapy and coronary revascularization in end-stage renal failure. Herz.

[5] Zinman B, Wanner C, Lachin JM (2015). Empagliflozin, cardiovascular outcomes, and mortality in type 2 diabetes. N Engl J Med.

[6] Wanner C, Inzucchi SE, Lachin JM (2016). Empagliflozin and progression of kidney disease in type 2 diabetes. N Engl J Med.

[7] Packer M, Anker SD, Butler J (2020). Cardiovascular and renal outcomes with empagliflozin in heart failure. N Engl J Med.

[8] Neal B, Perkovic V, Mahaffey KW (2017). Canagliflozin and cardiovascular and renal events in type 2 diabetes. N Engl J Med.

[9] Perkovic V, Jardine MJ, Neal B (2019). Canagliflozin and renal outcomes in type 2 diabetes and nephropathy. N Engl J Med.

[10] Wiviott SD, Raz I, Bonaca MP (2018). Dapagliflozin and cardiovascular outcomes in type 2 diabetes. N Engl J Med.

[11] McMurray JJV, Solomon SD, Inzucchi SE (2019). Dapagliflozin in patients with heart failure and reduced ejection fraction. N Engl J Med.

[12] Heerspink HJL, Stefánsson BV, Correa-Rotter R (2020). Dapagliflozin in patients with chronic kidney disease. N Engl J Med.

[13] Bhatt DL, Szarek M, Pitt B (2021). Sotagliflozin in patients with diabetes and chronic kidney disease. N Engl J Med.

[14] Al Dhaybi O, Bakris G (2017). Mineralocorticoid antagonists in chronic kidney disease. Curr Opin Nephrol Hypertens.

[15] Bakris GL, Agarwal R, Anker SD (2020). Effect of finerenone on chronic kidney disease outcomes in type 2 diabetes. N Engl J Med.

[16] KDIGO (2020) Hypertension guidelines. https://kdigo.org/wp-content/uploads/2016/10/KDIGO. Zugegriffen: 14. Feb. 2021

[17] Cheung AK, Rahman M, Reboussin DM (2017). Effects of intensive BP control in CKD. J Am Soc Nephrol.

[18] Wright JT, Williamson JD, Whelton PK (2015). SPRINT Trial. N Engl J Med.

[19] Cushman WC, Evans Evans GW, Byington RP, ACCORD Study Group (2010). Effects of intensive blood-pressure control in type 2 diabetes mellitus. N Engl J Med.

[20] Whelton PK, Carey RM, Aronow WS (2018). 2017 ACC/AHA/AAPA/ABC/ACPM/AGS/APhA/ASH/ASPC/NMA/PCNA Guideline for the prevention, detection, evaluation, and management of high blood pressure in adults: a report of the American College of Cardiology/American Heart Association Task Force on clinical practice guidelines. Circulation.

[21] Williams B, Mancia G, Spiering W (2018). 2018 ESC/ESH Guidelines for the management of arterial hypertension. Eur Heart J.

[22] Emrich IE, Böhm M, Mahfoud F (2019). The 2018 ESC/ESH guidelines for the management of arterial hypertension: a German point of view. Eur Heart J.

[23] Qiao Y, Shin JI, Chen TK (2020). Association between renin-angiotensin system blockade discontinuation and all-cause mortality among persons with low estimated glomerular filtration rate. JAMA Intern Med.

[24] Patel KK, Shah SY, Arrigain S (2019). Characteristics and outcomes of patients with aortic stenosis and chronic kidney disease. J Am Heart Assoc.

[25] Cubeddu RJ, Asher CR, Lowry AM (2020). Impact of transcatheter aortic valve replacement on severity of chronic kidney disease. J Am Coll Cardiol.

[26] Armijo G, Estevez-Loureiro R, Carrasco-Chinchilla F (2020). Acute kidney injury after percutaneous edge-to-edge mitral repair. J Am Coll Cardiol.

[27] Ronco C, Bellomo R, Kellum JA (2019). Acute kidney injury. Lancet.

[28] Gaudry S, Hajage D, Benichou N (2020). Delayed versus early initiation of renal replacement therapy for severe acute kidney injury: a systematic review and individual patient data meta-analysis of randomised clinical trials. Lancet.

[29] Gaudry S, Hajage D, Schortgen F (2016). Initiation strategies for renal-replacement therapy in the intensive care unit. N Engl J Med.

[30] Barbar SD, Clere-Jehl R, Bourredjem A (2018). Timing of renal-replacement therapy in patients with acute kidney injury and sepsis. N Engl J Med.

[31] The STARRT-AKI Investigators for the Canadian Critical Care Trials Group, Australian and New Zealand Intensive Care Society Clinical Trials Group, United Kingdom Critical Care Research Group, Canadian Nephrology Trials Network,, Irish Critical Care Trials Group (2020). Timing of initiation of renal-replacement therapy in acute kidney injury. N Engl J Med.

[32] Bangalore S, Maron DJ, O’Brien SM (2020). Management of coronary disease in patients with advanced kidney disease. N Engl J Med.

